# Activation of Serotonin 5-HT_7_ Receptors Modulates Hippocampal Synaptic Plasticity by Stimulation of Adenylate Cyclases and Rescues Learning and Behavior in a Mouse Model of Fragile X Syndrome

**DOI:** 10.3389/fnmol.2018.00353

**Published:** 2018-10-02

**Authors:** Lara Costa, Lara Maria Sardone, Carmela Maria Bonaccorso, Simona D’Antoni, Michela Spatuzza, Walter Gulisano, Maria Rosaria Tropea, Daniela Puzzo, Marcello Leopoldo, Enza Lacivita, Maria Vincenza Catania, Lucia Ciranna

**Affiliations:** ^1^Department of Clinical and Experimental Medicine, University of Messina, Messina, Italy; ^2^Department of Biomedical and Biotechnological Sciences, University of Catania, Catania, Italy; ^3^Oasi Research Institute, IRCCS, Troina, Italy; ^4^Institute of Neurological Sciences (ISN), National Research Council (CNR), Catania, Italy; ^5^Department of Pharmacy — Drug Sciences, University of Bari, Bari, Italy

**Keywords:** serotonin, 5-HT_7_ receptor, fragile X syndrome, cyclic AMP, mGluR-LTD, learning, PACAP

## Abstract

We have previously demonstrated that activation of serotonin 5-HT_7_ receptors (5-HT_7_R) reverses metabotropic glutamate receptor-mediated long term depression (mGluR-LTD) in the hippocampus of wild-type (WT) and *Fmr1* Knockout (KO) mice, a model of Fragile X Syndrome (FXS) in which mGluR-LTD is abnormally enhanced. Here, we have investigated intracellular mechanisms underlying the effect of 5-HT_7_R activation using patch clamp on hippocampal slices. Furthermore, we have tested whether *in vivo* administration of LP-211, a selective 5-HT_7_R agonist, can rescue learning and behavior in *Fmr1* KO mice. In the presence of an adenylate cyclase blocker, mGluR-LTD was slightly enhanced in WT and therefore the difference between mGluR-LTD in WT and *Fmr1* KO slices was no longer present. Conversely, activation of adenylate cyclase by either forskolin or Pituitary Adenylate Cyclase Activating Polypeptide (PACAP) completely reversed mGluR-LTD in WT and *Fmr1* KO. 5-HT_7_R activation reversed mGluR-LTD in WT and corrected exaggerated mGluR-LTD in *Fmr1* KO; this effect was abolished by blockade of either adenylate cyclase or protein kinase A (PKA). Exposure of hippocampal slices to LP-211 caused an increased phosphorylation of extracellular signal regulated kinase (ERK), an intracellular effector involved in mGluR-LTD, in WT mice. Conversely, this effect was barely detectable in *Fmr1* KO mice, suggesting that 5-HT_7_R-mediated reversal of mGluR-LTD does not require ERK stimulation. Finally, an acute *in vivo* administration of LP-211 improved novel object recognition (NOR) performance in WT and *Fmr1* KO mice and reduced stereotyped behavior in *Fmr1* KO mice. Our results indicate that mGluR-LTD in WT and *Fmr1* KO slices is bidirectionally modulated in conditions of either reduced or enhanced cAMP formation. Activation of 5-HT_7_ receptors reverses mGluR-LTD by activation of the cAMP/PKA intracellular pathway. Importantly, a systemic administration of a 5-HT_7_R agonist to *Fmr1* KO mice corrected learning deficits and repetitive behavior. We suggest that selective 5-HT_7_R agonists might become novel pharmacological tools for FXS therapy.

## Introduction

Fragile X Syndrome (FXS), the most common single-gene cause of intellectual disability, autism and epilepsy (Garber et al., 2008), is caused by transcriptional silencing of the FMR1 gene coding for Fragile X Mental Retardation Protein (FMRP), an mRNA-binding protein that regulates translation of several synaptic proteins (Pfeiffer and Huber, 2009). An abnormal morphology and density of dendritic spines was observed in the brain cortex of FXS patients (Irwin et al., 2001) and in the cortex and hippocampus of *Fmr1* knockout (KO) mice (Comery et al., 1997; Nimchinsky et al., 2001; Grossman et al., 2010), a model of FXS, suggesting a dysfunction of excitatory synaptic transmission in several brain regions. Accordingly, studies on the mouse model of FXS revealed altered synaptic plasticity mediated by metabotropic glutamate receptors (mGluRs; Pfeiffer and Huber, 2009). Metabotropic glutamate receptor-mediated long term depression (mGluR-LTD), a form of plasticity playing a crucial role in cognition and in behavioral flexibility (Luscher and Huber, 2010; Sanderson et al., 2016), is pathologically enhanced in the hippocampus of *Fmr1* KO mice (Huber et al., 2002) and is regarded as the electrophysiological readout of synaptic malfunction in the mouse model of FXS (Bear et al., 2004; Waung and Huber, 2009).

In the last few years, a decrease of cyclic AMP (cAMP) has been proposed to be involved in FXS pathogenesis (Kelley et al., 2008). Different data support a “cAMP theory” of FXS: early observations indicated a reduction of basal cAMP levels in blood platelets from FXS patients and a decrease in cAMP production following adenylate cyclase stimulation (Berry-Kravis and Huttenlocher, 1992; Berry-Kravis and Sklena, 1993). Reduced cAMP production was later detected in the brain of *dfmr1* null drosophila, in brain and blood platelets of *Fmr1* KO mice and in neural precursor cells from human FXS fetal tissues (Kelley et al., 2007). Besides, pharmacological manipulation with agents that potentially increase cAMP, i.e., inhibitors of group II mGluRs and phosphodiesterase IV inhibitors (PDE4-Is), reversed the mGluR-LTD alteration in FXS mouse models (Choi et al., 2011, 2015, 2016), leading to the hypothesis that increasing cAMP formation might become a potential therapeutic strategy to rescue FXS phenotype.

We have previously demonstrated that exaggerated mGluR-LTD in *Fmr1* KO mice was reversed by activation of 5-HT_7_ receptors (5-HT_7_Rs) for serotonin (Costa et al., 2012a, 2015). 5-HT_7_Rs are positively coupled to adenylate cyclase and are highly expressed in the hippocampus, where they are believed to regulate learning and memory (Matthys et al., 2011; Ciranna and Catania, 2014).

In the present work, we have tested the hypothesis that a dysregulation of cAMP pathway might play a role in abnormal mGluR-LTD in *Fmr1* KO hippocampal neurons. In this perspective, we evaluated whether the activation of 5-HT_7_Rs, coupled to Gs, rescues mGluR-LTD by increasing cAMP levels. We also tested whether *in vivo* administration of a 5-HT_7_R agonist could rescue learning ability and the behavioral phenotype in a mouse model of FXS.

## Materials and Methods

### Animals

All experiments were performed in mice obtained from a breeding colony kept at the University of Catania. We used *Fmr1* KO mice and wild-type (WT) littermates from C57BL/6J strain for electrophysiology and behavioral experiments; both FVB and C57BL/6J strains were used for Western blotting. We crossed homozygous *Fmr1* KO females with hemizygous *Fmr1* KO males and both male and female pups were used in our experiments. Mice were maintained with a controlled temperature (21°C ± 1°C) and humidity (50%) on a 12 h light/dark cycle, with *ad libitum* food and water. All animal experimentation was conducted in accordance with the European Community Council guidelines (2010/63/EU) and was approved by the University Institutional Animal Care and Use Committee (Projects #181; 250—approval number: 352/2016-PR, Project #286—approval number: 174/2017-PR).

### Electrophysiology

Acute hippocampal slices were prepared as previously described (Costa et al., 2012b) from WT and *Fmr1* KO mice on a C57BL/6J background [postnatal (PN) age 14–23 days]. The brains were removed, placed in oxygenated ice-cold artificial cerebrospinal fluid (ACSF; in mM NaCl 124; KCl 3.0; NaH_2_PO_4_ 1.2; MgSO_4_ 1.2; CaCl_2_ 2.0; NaHCO_3_ 26; D-glucose 10, pH 7.3) and cut into 300 μm slices with a vibratome (Leica VT 1200S). Slices were continually perfused with oxygenated ACSF and viewed with infrared microscopy (Leica DMLFS). Schaffer collaterals were stimulated with negative current pulses (duration 0.3 ms, delivered every 15 s by A310 Accupulser, WPI, USA). Evoked excitatory post synaptic currents (EPSCs) were recorded under whole-cell from CA1 pyramidal neurons (holding potential −70 mV; EPC7-plus amplifier HEKA, Germany). Stimulation intensity was set to induce half-maximal EPSC amplitude. Series resistance (Rs) was continuously monitored by 10 mV hyperpolarizing pulses; recordings were discarded from analysis if Rs changed by more than 20%. EPSC traces were filtered at 3 kHz and digitized at 10 kHz. Data were acquired and analyzed using Signal software (CED, England). The recording micropipette (resistance 1.5–3 MΩ) was filled with intracellular solution (in mM: K-gluconate 140; HEPES 10; NaCl 10; MgCl2 2; EGTA 0.2; Mg-ATP 3.5; Na-GTP 1; pH 7.3).

To isolate AMPA receptor-mediated EPSCs, bath solution (ACSF; flow rate of 1.5 ml/min) routinely contained (-)-bicuculline methiodide (5 μM, Hello Bio) and D-(-)-2-Amino-5-phosphonopentanoic acid (D-AP5, 50 μM, Hello Bio).

(S)-3,5-dihydroxyphenylglycine (DHPG; 100 μM; Hello Bio), forskolin (20 μM; Tocris), PACAP-38 (PACAP, 10 nM, Tocris) and LP-211 (10 nM) were dissolved in ACSF and applied by bath perfusion. LP-211 was synthesized and provided by the research group of Prof. Leopoldo (University of Bari, Italy). SQ-22536 (10 μM, Tocris), protein kinase A (PKA) inhibitor fragment 6–22 (PKI; 20 μM, Tocris) and PD-98059 (40 μM, Tocris) were included in the intracellular solution.

### LTD Data Analysis

Peak amplitude values of EPSCs were averaged over 1 min and expressed as % of baseline EPSC amplitude (calculated from EPSCs recorded during at least 15 min before DHPG application). % EPSC values from groups of neurons were pooled (mean ± standard error of mean, SEM) and graphically represented as a function of time. For each neuron studied, the amount of mGluR-LTD was calculated by averaging EPSC values recorded during 5 min between 40 min and 45 min after LTD induction and was expressed as percentage of baseline (% EPSC amplitude). Cumulative bar graphs indicate % EPSC amplitude (mean ± SEM from groups of neurons) after application of DHPG alone (control LTD) or DHPG with the 5-HT_7_ receptor agonist under different experimental conditions. EPSC amplitude values from two groups of neurons were compared using unpaired Student’s *t*-test, with *n* indicating the number of neurons tested in each condition; several groups of data were compared by one-way ANOVA followed by Tukey’s multiple comparisons test (GraphPad Prism 6, La Jolla, CA, USA).

### Western Blotting

We used brains of WT (FVB and C57BL6J strains) and *Fmr1* KO (C57BL6J strains) mice for stimulation assay. Hippocampi from mice at PN age 14–23 days were dissected, quickly cut into 350 μm slices using a McIlwain tissue chopper and transferred to oxygenated ice-cold artificial cerebrospinal fluid (ACSF; in mM NaCl 124, KCl 3, CaCl_2_ 2, NaHCO_3_ 25, NaH_2_PO_4_ 1.1, MgSO_4_ 2, D-glucose 10, pH 7.4) containing protease inhibitor cocktails EDTA free (Roche) and (+)-MK 801 maleate (1 μM; Tocris). Thereafter, slices were pre-incubated with ACSF containing protease inhibitor cocktails EDTA free (Roche) and (+)-MK 801 maleate (1 μM; Tocris) for 35 min at 32°C. Then, slices were exposed to LP-211 (10 nM) for 5 min; when present, H-89 dihydrochloride (1 μM; Tocris) was added 5 min before stimulation with LP-211 and maintained during LP-211 stimulation. Then, slices were washed three times in ice-cold ACSF and immediately frozen at −80°C until use.

Frozen slices were homogenized in ice-cold extraction buffer [Tris 50 mM pH 8; NaCl 150 mM; MgCl_2_ 1.5 mM; NP40 1%; protease inhibitor cocktail EDTA free and phosphatase inhibitor cocktail (Roche)], and centrifuged for 30 min at 18,000× *g* at 4°C. The supernatant was collected and one aliquot was used for protein determination using the BCA methods (Pierce BCA protein assay kit). 80–100 μg of proteins were eluted with SDS sample buffer and separated onto 9% SDS-PAGE, as previously described (Bonaccorso et al., 2015). The following primary antibodies were used for Western blotting: polyclonal rabbit p44/42 MAP-kinase antibody (1:1,000, Cell Signaling), polyclonal rabbit phospho-p44/42 MAP-kinase antibody (1:1,000, Cell Signaling), while polyclonal rabbit GAPDH antibody (1:1,000, Cell Signaling) was used as a loading control.

Blots were developed by using the specific Western Breeze Chemiluminescent Immunodetection Kit (Invitrogen). Band densities were measured using ImageJ 1.49 software (Figure 5; Supplementary Figure S2) or the VersaDoc 4000 Imaging System (Bio-Rad, Figure 5; Supplementary Figure S3). The expression levels of phospho-extracellular signal regulated kinase (ERK)1/2 and ERK1/2 were normalized first against the levels of respective GAPDH and then calculated as ratio of total ERK signal.

**Figure 1:**
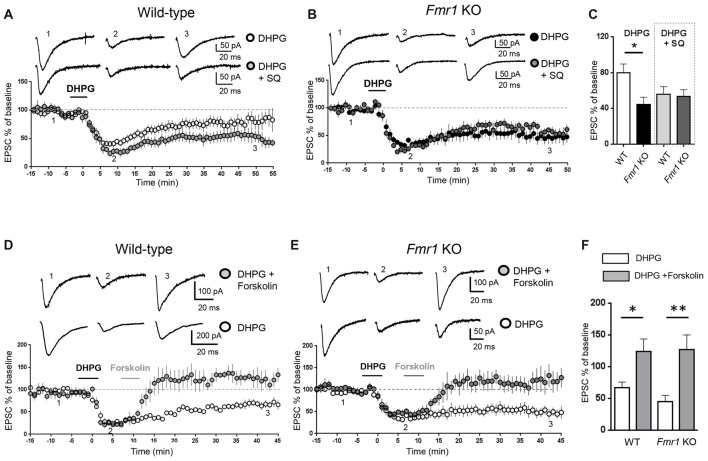
Modulation of adenylate cyclase activity modified metabotropic glutamate receptor-mediated long term depression (mGluR-LTD) in wild-type (WT) and *Fmr1* Knockout (KO) slices. AMPAR-mediated excitatory post-synaptic currents (EPSCs) were recorded in the presence of D-AP5 (50 μM) and bicuculline (5 μM) under whole-cell patch clamp in the CA3–CA1 synapse in hippocampal slices from WT and *Fmr1* KO mice. **(A)** In WT slices, bath application of the group I mGluR agonist (S)-3,5-dihydroxyphenylglycine (DHPG; 100 μM, 5 min) induced a long-term depression (mGluR-LTD) of EPSC amplitude (white dots, *n* = 11). When the adenylate cyclase blocker SQ 22536 (SQ, 10 μM) was added to intracellular medium, DHPG-induced mGluR-LTD was slightly enhanced (gray dots, *n* = 8) with respect to control. Individual representative EPSC traces are shown on top of the graph (1: baseline; 2: acute EPSC reduction; 3: mGluR-LTD). **(B)** In *Fmr1* KO slices, DHPG-induced mGluR-LTD (black dots, *n* = 9) was enhanced with respect to WT (**A**, white dots) and was not further enhanced in the presence of intracellular SQ 22536 (SQ, 10 μM; dark gray dots, *n* = 6). **(C)** The bar graph shows the amount of mGluR-LTD (mean EPSC amplitude in all tested neurons, expressed as % of baseline EPSC amplitude). The amount of mGluR-LTD was compared by one-way ANOVA followed by Tukey’s multiple comparisons test in the four experimental conditions illustrated in **(A,B)** (*P = 0.03). In control conditions (DHPG), mGluR-LTD in *Fmr1* KO was significantly enhanced with respect to WT (*P = 0.025, by unpaired *t*-test). In the presence of intracellular SQ 22536 (DHPG+SQ), the amount of mGluR-LTD in WT was not significantly different from Fmr1 KO (*P* = 0.83, by unpaired *t*-test) and was also comparable to that observed in Fmr1 KO in control conditions (*P* = 0.35 by unpaired *t*-test). **(D)** When DHPG application was followed by bath application of forskolin, a direct activator of adenylate cyclase (20 μM, 5 min), mGluR-LTD was reduced (gray dots, *n* = 4) with respect to control conditions (white dots; *n* = 5). **(E)** The same result was observed in *Fmr1* KO slices: DHPG-induced mGluR-LTD (white dots; *n* = 9) was completely reversed by application of forskolin (20 μM, 5 min; gray dots, *n* = 5). **(F)** Reversal of mGluR-LTD by forskolin was statistically significant both in WT (*P = 0.025; by unpaired *t*-test) and in *Fmr1* KO (**P = 0.0026).

**Figure 2:**
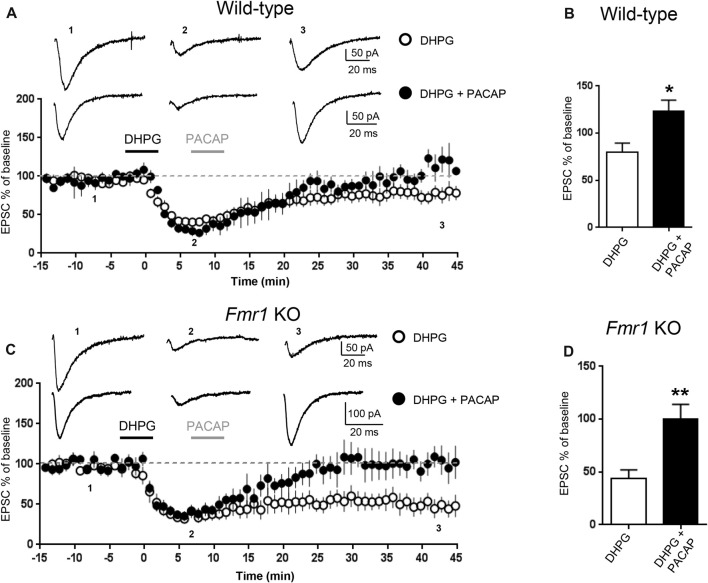
Pituitary adenylate cyclase activating peptide (PACAP) reversed mGluR-LTD in WT and in *Fmr1* KO hippocampus. mGluR-LTD was induced by application of DHPG (100 μM, 5 min). Application of PACAP (10 nM, 5 min) fully reversed DHPG-induced mGluR-LTD in WT **(A)** and in *Fmr1* KO slices **(C)**. **(B,D)** Reversal of mGluR-LTD by PACAP was statistically significant both in WT (*P = 0.04, by unpaired *t*-test) and in *Fmr1* KO slices (**P = 0.0025, unpaired *t*-test).

**Figure 3:**
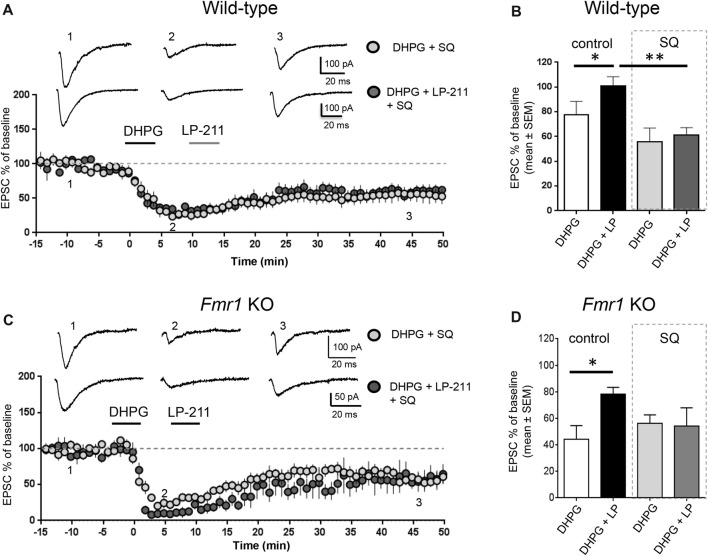
Activation of 5-HT_7_ receptors reversed mGluR-LTD by stimulation of adenylate cyclase. **(A)** In hippocampal WT slices, application of LP-211 did not modify mGluR-LTD in the presence of the adenylate cyclase blocker SQ 22536 (10 μM, included in intracellular pipette solution; dark gray dots, *n* = 6).** (B)** The bar graph shows the amount of mGluR-LTD measured 45 min after LTD induction (mean EPSC amplitude in all tested neurons, expressed as % of baseline EPSC amplitude). In WT slices, bath applications of DHPG-induced mGluR-LTD (white column; *n* = 11) that was completely reversed when DHPG application was followed by application of the 5-HT_7_R agonist LP-211 (10 nM, 5 min; black column, *n* = 7; *P = 0.03 by unpaired *t*-test). In the presence of intracellular SQ 22536 (10 μM; gray column, *n* = 6), the amount of mGluR-LTD was slightly increased with respect to control and was not reversed by application of LP-211 (10 nM, 5 min; dark gray column, *n* = 6; significantly different from the effect of LP-211 in control conditions, **P = 0.0026 by unpaired *t*-test). **(C)** Similarly, in *Fmr1* KO slices application of LP-211 (10 nM, 5 min) had no effect on mGluR-LTD in the presence of intracellular SQ 22536 (10 μM; dark gray column, *n* = 6). **(D)** In *Fmr1* KO slices, application of LP-211 reversed DHPG-induced mGluR-LTD only in control conditions (black column, *n* = 6; *P = 0.02 by unpaired *t*-test) but not in the presence of SQ 22536 (SQ, 10 μM; gray column, *n* = 6), showing that 5-HT_7_R-mediated effect required stimulation of adenylate cyclase also in *Fmr1* KO slices.

**Figure 4:**
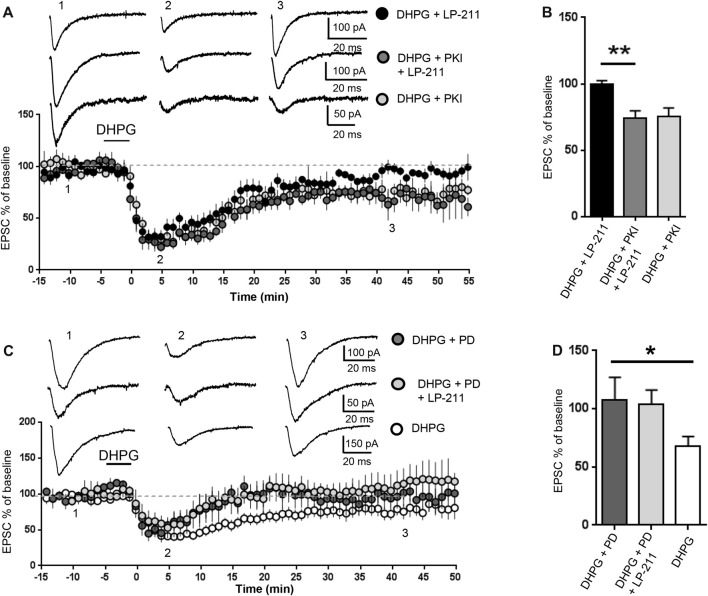
5-HT_7_ receptor-mediated reversal of mGluR-LTD required protein kinase A (PKA) and was occluded by inhibition of extracellular signal regulated kinase (ERK). mGluR-LTD was induced by bath application of DHPG (100 μM, 5 min) in WT slices in control conditions and in the presence of peptide fragment 6–22 (PKI, added to intracellular solution), an inhibitor of PKA. **(A)** Application of LP-211 (10 nM, 5 min) reversed mGluR-LTD in control conditions (black dots, *n* = 7) but had no effect in the presence of PKI (dark gray dots, *n* = 5). **(B)** In the presence of PKI, the effect of LP-211 was significantly reduced (**P = 0.0013, by unpaired *t*-test), indicating that PKA activation was necessary for 5-HT_7_R-mediated reversal of mGluR-LTD.** (C)** mGluR-LTD was induced by bath application of DHPG (100 μM, 5 min) in WT slices in control conditions (white dots, *n* = 11) and in the presence of the mitogen-activated protein kinase (MAPK)/ERK blocker PD-98059 (PD, 40 μM, added to intracellular solution). Intracellular PD-98059 completely reversed mGluR-LTD (dark gray dots, *n* = 5) and occluded the effect of LP-211 (10 nM, 5 min; gray dots, *n* = 5). **(D)** mGluR-LTD was significantly reversed by PD-98059 (*P = 0.03) and was not further modified by LP-211 (*P* = 0.52).

**Figure 5:**
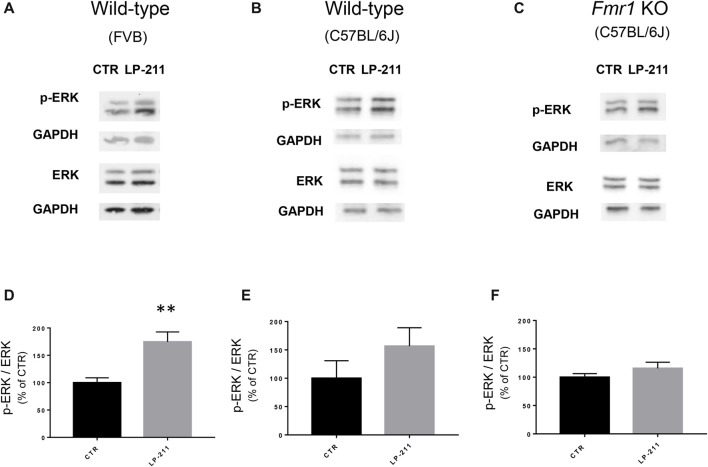
Activation of 5-HT_7_ receptors stimulated ERK phosphorylation. **(A–C)** Representative immunoblots showing the levels of phosphorylated and total ERK1/2 in control and LP-211 treated (10 nM, 5 min) hippocampal slices from WT (FVB strain; **A**), WT (C57BL/6J strain; **B**), and Fmr1 KO (C57BL/6J strain; **C**) mice. **(D–F)** Semi-quantitative analysis of phosphorylated ERK1/2 vs. total ERK1/2 in control and LP-211 treated hippocampal slices from WT (FVB strain; **D**), WT (C57BL/6J strain; **E**), and Fmr1 KO (C57BL/6J; **F**) mice. GAPDH was used as loading control. Relative optical density is presented as percentage of control. Data represent mean ± SEM of five **(D)**, four **(E,F)** separate experiments, each performed on a pool of three mice. **p = 0.0064 by unpaired *t*-test. Full length immunoblots are shown in Supplementary Figures S2, S3.

### Behavior

Novel object recognition (NOR) test was performed as previously described (Puzzo et al., 2013) on sex-balanced WT and *Fmr1* KO mice (C57BL/6J background; age 3–4 months). After 3 days of habituation (10 min/day), mice underwent the training session (T1). They were placed in the arena for 10 min, a time sufficient to learn the task, and allowed to explore two identical objects, i.e., two glass beakers upside-down placed in the central part of the box, equally distant from the perimeter. Thirty minutes before T1 they received a i.p. injection of LP-211 (3 mg/Kg) or vehicle. Twenty-four hours after T1 mice underwent the second trial (T2) where a “familiar” (i.e., the one used for T1) and a “novel” object (ceramic cup) were presented to test memory retention. The novel object was placed on the left or the right side of the box in a randomly but balanced manner, to minimize potential biases because of a preference for particular locations or objects. To avoid olfactory cues, the objects and the apparatus were cleaned with 70% ethanol after each trial. Animal exploration, defined as the mouse pointing its nose toward the object from a distance not >2 cm (as marked by a reference circle), was evaluated in T2. We analyzed: (i) % exploration of the novel and % exploration of the familiar object; (ii) discrimination (D) index calculated as “exploration of novel object minus exploration of familiar object/total exploration time”; (iii) latency to first approach to novel object; and (iv) total exploration time.

Marble burying test was performed as previously described (Thomas et al., 2009), under standard room lighting and noise conditions. Twenty green glass marbles (15 mm in diameter) were arranged in a clean standard cage filled with a sani-chips bedding in a 4 × 5-cm pattern. Each mouse was gently placed into the cage and allowed to explore for 20 min. The number of marbles buried (covered by >50% bedding) was recorded.

Open field (OF) was performed as previously described (Palmeri et al., 2016). Each mouse was gently put in the arena (a white plastic bow divided into sectors by black lines) and was allowed to freely explore the environment for 5 min. The test was performed in a quiet, darkened room and one light bulb provided a bright illumination. We scored the following parameters: (i) % time spent into the center; (ii) number of entries into the center; (iii) “horizontal activity”, time spent moving into the arena; (iv) rearing or “vertical activity”, time spent erected on its hind legs; (v) grooming (time spent scratching itself with the forepaws); (vi) freezing (time of immobility); and (vii) defecation (number of fecal boli produced).

## Results

### Modulation of Adenylate Cyclase Activity Modified the Amount of mGluR-LTD in WT and *Fmr1* KO Hippocampus

Excitatory post-synaptic currents (EPSCs) mediated by AMPA receptors were recorded in the CA3-CA1 synapse on hippocampal slices from WT and *Fmr1* KO mice. mGluR-LTD of EPSCs was chemically induced by bath application of the mGluR agonist DHPG (100 μM, 5 min). We have previously confirmed (Costa et al., 2012a) that mGluR-LTD in *Fmr1* KO slices is enhanced compared to WT, consistent with previous findings (Huber et al., 2002). To test if changes in intracellular cAMP levels might affect mGluR-LTD, we measured the amount of mGluR-LTD in hippocampal slices from WT and *Fmr1* KO mice under experimental conditions reducing or enhancing cAMP levels in the recorded neuron. When the adenylate cyclase inhibitor SQ 22536 (10 μM) was included in the intracellular pipette solution (Figure 1A), the amount of mGluR-LTD in WT slices showed a trend towards an enhancement compared to control conditions, although not statistically significant (EPSC % amplitude measured 45 min after LTD induction: 79.5 ± 10, vs. 56 ± 9 comparing control vs. SQ 22536, *n* = 11/7; unpaired *t*-test: *t*_(16)_ = 1.56; *P* = 0.07).

In *Fmr1* KO slices, the amount of mGluR-LTD in control conditions was significantly higher than in WT (EPSC %: 44 ± 8 vs. 79.5 ± 10, comparing *Fmr1* KO vs. WT, *n* = 9/11; *t*_(18)_ = 2.44; *P* = 0.025; Figure 1C) and was not further enhanced by the adenylate cyclase blocker (EPSC %: 44 ± 8 vs. 53 ± 7, comparing *Fmr1* KO control vs. *Fmr1* KO + SQ 22536, *n* = 9/6; *t*_(13)_ = 0.76; *P* = 0.45; Figures 1B,C).

When comparing the amount of mGluR-LTD in all the different experimental conditions (Figure 1C), a significant difference was found only between WT and *Fmr1* KO in control conditions (*P* = 0.03 by one-way ANOVA followed by Tukey’s multiple comparisons). In the presence of SQ 22536, the amount of mGluR-LTD was comparable in WT and *Fmr1* KO slices (EPSC %: 55 ± 10 vs. 53 ± 7 comparing WT + SQ 22536 vs. *Fmr1* KO + SQ 22536, *n* = 7/6; *t*_(11)_ = 0.21; *P* = 0.83) and was not significantly different from that measured in *Fmr1* KO slices without SQ 22536 (*t*_(14)_ = 0.96; *P* = 0.35).

Conversely, a direct stimulation of adenylate cyclase by bath application of forskolin (20 μM, 5 min) completely abolished DHPG-induced mGluR-LTD in both WT (EPSC %: 67 ± 8 vs. 124 ± 20, *n* = 5/4; *t*_(7)_ = 2.8; *P* = 0.025, comparing DHPG vs. DHPG + forskolin, Figures 1D,F) and *Fmr1* KO neurons (EPSC %: 45 ± 10 vs. 127 ± 23, *n* = 9/5; *t*_(12)_ = 3.7; *P* = 0.0026, comparing DHPG vs. DHPG + forskolin, Figures 1E,F), showing that mGluR-LTD was reversed by increasing cAMP levels.

To further study the effect of increasing cAMP levels on mGluR-LTD, we tested the effects of Pituitary Adenylate Cyclase Activating Polyeptide (PACAP), a potent endogenous stimulator of adenylate cyclase activity (Harmar et al., 2012). Application of PACAP (10 nM, 5 min) reversed DHPG-induced mGluR-LTD both in WT (EPSC% 79.5 ± 10 vs. 123 ± 11, DHPG vs. DHPG + PACAP, *n* = 11/6, *t*_(15)_ = 2.72, *P* = 0.015, Figures 2A,B) and in *Fmr1* KO hippocampus (EPSC% 44 ± 8 vs. 100 ± 14, DHPG vs. DHPG + PACAP, *n* = 9/7, *t*_(13)_ = 3.66, *P* = 0.0025, Figures 2C,D).

### Activation of 5-HT_7_ Receptors Reversed mGluR-LTD by Stimulation of Adenylate Cyclase and Protein Kinase A

We tested the effect of LP-211, a 5-HT_7_R agonist, on mGluR-LTD in the presence of pharmacological modulators of the cAMP/PKA pathway. LP-211 was applied at 10 nM dose in order to activate selectively 5-HT_7_ receptors without activating the 5-HT_1A_ subtype, based on its reported binding affinity for 5-HT_7_ and 5-HT_1A_ receptors (Ki 0.58 and 188 nM respectively, see compound 25 in Leopoldo et al. (2008). In the presence of intracellular SQ 22536 (10 μM), application of LP-211 did not modify the amount of mGluR-LTD (EPSC %: 55 ± 10 vs. 61 ± 6, *n* = 7/6; *t*_(11)_ = 0.39, *P* = 0.69 comparing DHPG + SQ 22536 vs. DHPG + SQ 22536 + LP-211; Figures 3A,B). Therefore, in the presence of SQ 22536 we did not observe the 5-HT7R-mediated reversal of mGluR-LTD that we described in control conditions (Costa et al., 2012a, 2015).

Similar to WT, in *Fmr1* KO 5-HT_7_R-mediated reversal of mGluR-LTD was abolished by SQ-22536 (EPSC %: 53 ± 7 vs. 54 ± 13, *n* = 6/5; *t*_(9)_ = 0.04; *P* = 0.96, comparing DHPG + SQ22536 vs. DHPG + SQ22536 + LP-211; Figures 3C,D), showing that reversal of mGluR-LTD by 5-HT_7_R activation was mediated by cAMP.

We then tested a possible involvement of PKA, one of the main cAMP effector targets (Taylor et al., 1990). The PKA inhibitor peptide fragment 6–22 (PKI, 20 μM) was added to the intracellular solution (Figures 4A,B): in this condition, the amount of DHPG-induced mGluR-LTD was not significantly different from control (EPSC % amplitude: 75 ± 6 vs. 79.5 ± 10, *n* = 10/11; *t*_(19)_ = 0.32; *P* = 0.75, comparing PKI vs. control) but the effect of LP-211 was significantly reduced (EPSC %: 74 ± 5 vs. 99.5 ± 3, *n* = 6/7; *t*_(11)_ = 4.26; *P* = 0.0013, comparing LP-211+PKI vs. LP-211; Figures 4A,B), indicating an involvement of PKA in 5-HT_7_R-mediated reversal of mGluR-LTD.

### 5-HT_7_R-Activation Stimulated Extracellular Signal-Regulated Kinase (ERK)

The cAMP pathway can also interact with the RAS/MEK/ERK signaling cascade (Dumaz and Marais, 2005), which is required for mGluR-LTD and dysregulated in the mouse model of FXS (Hou et al., 2006; Kim et al., 2008; Osterweil et al., 2010; Sawicka et al., 2016). Intracellular PD-98059 (40 μM), a mitogen-activated protein kinase (MAPK)/ERK blocker, reversed mGluR-LTD in WT hippocampal slices: the amount of mGluR-LTD was significantly decreased compared to control conditions (EPSC% 25 min after LTD induction: 68±8 vs. 108±19, *n* = 11/5; *t*_(14)_ = 2.27; *P* = 0.03; control vs. PD-98059; Figures 4A,B). This result is consistent with previous data showing that ERK activation is necessary for mGluR-LTD (Gallagher et al., 2004).

mGluR-LTD was equally reversed by LP-211 (10 nM, 5 min) in the presence of PD-98059 (40 μM) and by PD-98059 alone (EPSC % amplitude: 112 ± 20 vs. 96 ± 11; *n* = 5/5; *t*_(8)_ = 0.66; *P* = 0.52, comparing LP-211+PD-98059 vs. PD-98059; Figures 4C,D), indicating that PD-98059 occluded the effect of LP-211. Therefore, using mGluR-LTD as readout of 5-HT_7_R-mediated effect, we could not test if 5-HT_7_R activation was modulating ERK activity.

To check for a possible coupling of 5-HT_7_Rs to the ERK pathway, we measured ERK phosphorylation levels by Western blotting. Exposure of hippocampal slices to LP-211 (10 nM) for 5 min increased ERK1/2 phosphorylation, showing that 5-HT_7_R activation stimulates ERK signaling (Figures 5A,B,D,E). Stimulation of ERK phosphorylation by LP-211 was observed in hippocampal slices from WT mice, although it resulted statistically significant only in WT of FVB strain (Figure 5D, FVB; CTR: 100 ± 14, LP-211: 182 ± 17, *n* = 5, *P* = 0.0064 by unpaired *t*-test; Figure 5E, C57BL/6J: CTR: 100 ± 31, LP-211: 157 ± 32.5, *n* = 4, *P* = 0.254 by unpaired *t-test*). An increased phosphorylation of both ERK1 and ERK2 was detected after LP-211 exposure (Supplementary Figure S1). Interestingly, LP-211 caused only a negligible increase of ERK1/2 phosphorylation levels in hippocampal slices from *Fmr1* KO mice (Figures 5C,F; CTR:100 ± 6; LP-211: 116 ± 11, *n* = 4, *P* = 0.255 by unpaired *t*-test). Overall, our data suggest that LP-211-mediated reversal of mGluR-LTD does not operate through stimulation of ERK signaling.

### Acute *in vivo* Administration of a 5-HT_7_R Agonist Improved Object Recognition Memory in WT and *Fmr1* KO Mice

**Figure 6:**
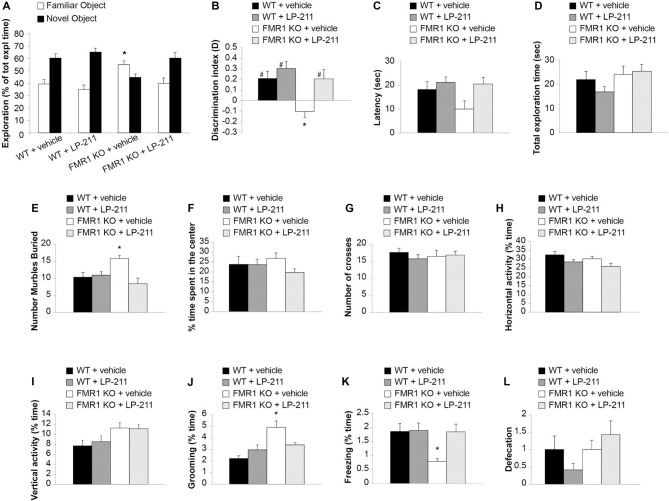
Acute *in vivo* administration of LP-211 improved memory and reduced stereotyped behavior in *Fmr1* KO mice. **(A)** Exploration times of familiar and novel object during T2 (after a 24-h retention interval) show that *Fmr1* KO mice treated with vehicle present an impairment of memory (higher amount of time exploring the familiar vs. the novel object; *p* < 0.0001) that is rescued by treatment with LP-211 (3 mg/kg 30 min before T1). WT + vehicle = 11; *Fmr1* KO + vehicle = 12; WT + LP-211 = 10; *Fmr1* KO + LP-211 = 10. **(B)** Analysis of the discrimination **(D)** index confirms that the impairment of recognition memory in *Fmr1* KO (*P* = 0.015 vs. WT) is rescued by LP-211 (*P* = 0.02 vs. *Fmr1* KO + LP-211). A difference from 0 is depicted with hashes (^#^*p* < 0.05)** (C)** Latency to first approach to the novel object and** (D)** total exploration time are comparable in the 4 groups of mice. **(E)** *Fmr1* KO treated with vehicle buried a higher number of marbles compared to WT (*P* = 0.002). This stereotypic behavior was rescued by treatment with LP-211 (3 mg/kg 30 min before test). WT + vehicle = 11; WT + LP-211 = 10; *Fmr1* KO + vehicle = 12; *Fmr1* KO + LP-211 = 10 for novel object recognition (NOR; **A–D**) and Marble Burying **(E)** tasks. **(F)** Time spent in the center of the arena and **(G)** the number of crosses into the center is comparable in the four groups of mice. **(H–I)** General locomotor activity analyzed as horizontal activity and vertical activity (rearing) is not modified by genotype and treatment. **(J)** The increase in stereotyped behavior such as grooming in *Fmr1* KO mice (*P* < 0.0001) is rescued by treatment with LP-211. **(K)** *Fmr1* KO mice show a decrease of spontaneous freezing behavior compared to WT littermates (*P* = 0.036) that is rescued by LP-211. **(L)** Defecation is comparable in the four groups of mice. WT + vehicle = 10; WT + LP-211 = 12; *Fmr1* KO + vehicle = 12; *Fmr1* KO + LP-211 = 14 for Open field (OF) test. Data are expressed as mean ± SEM. *Significant difference (*p* < 0.05 by one-way ANOVA with Bonferroni’s multiple comparisons test); ^#^difference with zero (one-sample *t*-test).

*Fmr1* KO mice show a cognitive impairment when evaluated by Novel object recognition (NOR) tasks based on the natural tendency of rodents to explore unfamiliar objects (Ventura et al., 2004; King and Jope, 2013; Franklin et al., 2014; Gomis-González et al., 2016). Here we first confirmed that *Fmr1* KO presented a damage of recognition memory, since their D index did not significantly differ from zero (*t*_(11)_ = 1.864, *P* = 0.089, *n* = 12; Figures 6A,B). Interestingly, *Fmr1* KO spent a higher amount of time exploring the old vs. the new object (55.18 ± 2.78 vs. 44.81 ± 2.78 s of exploration time familiar vs. novel object; paired *t*-test: *t*_(23)_ = 21.446, *P* < 0.0001; Figures 6A,B). WT littermates showed a normal recognition memory, as demonstrated by the higher time spent exploring the new object (39.61 ± 3.47 vs. 60.61 ± 3.47 s of exploration time familiar vs. novel object; *n* = 11; paired *t*-test: *t*_(21)_ = 15.039, *P* < 0.0001; Figure 6A) and D index different than zero (*t*_(10)_ = 2.99, *P* = 0.014; Figure 6B).

LP-211 is a brain-penetrant molecule, reaching the brain within 30 min after intraperitoneal injection in mice (Hedlund et al., 2010). Intraperitoneal treatment with LP-211 (3 mg/kg) 30 min before training rescued memory in *Fmr1* KO mice (39.74 ± 4.55 vs. 60.25 ± 4.44 s of exploration time familiar vs. novel object; *n* = 10; paired *t*-test: *t*_(19)_ = 12.804, *P* < 0.0001; Figure 6A; D: *t*_(9)_ = 2.308, *P* = 0.046; Figure 6B), without modifying cognitive performances in WT animals (34.93 ± 3.39 vs. 65.06 ± 3.39 s of exploration time familiar vs. novel object; *n* = 10; paired *t*-test: *t*_(19)_ = 14.344, *P* < 0.0001; D: *t*_(9)_ = 4.437, *P* = 0.002; Figures 6A,B). These findings were confirmed by the analyses of D among groups (one-way ANOVA with Bonferroni’s: *F*_(3,39)_ = 6.71, *P* = 0.001; WT vs. *Fmr1* KO *p* = 0.015; *Fmr1* KO vs. *Fmr1* KO + LP-211 *p* = 0.02). The 4 groups of mice did not show differences in the latency to first approach to the novel object (one-way ANOVA: *F*_(3,39)_ = 1.103, *P* = 0.360; Figure 6C) nor in total exploration time (one-way ANOVA: *F*_(3,39)_ = 1.394, *P* = 0.259; Figure 6D).

### Acute *in vivo* Administration of a 5-HT_7_R Agonist Reversed Stereotyped Behavior in *Fmr1* KO Mice

FXS patients present perseverative or stereotypic behaviors that can be studied in rodents through a marble burying task (Thomas et al., 2009). Here, we observed that *Fmr1* KO mice buried a higher number of marbles compared to WT littermates (Bonferroni’s *P* = 0.043; Figure 6E; Supplementary Figure S4), confirming previous data (Veeraragavan et al., 2012; Gholizadeh et al., 2014). An acute treatment with LP-211 (3 mg/kg, 30 min before trial) rescued this stereotypic behavior since it induced a reduction of marble burying in *Fmr1* KO mice (Bonferroni’s *P* = 0.002), whereas it did not affect WT behavior (Bonferroni’s *P* = 1; Figure 6E). ANOVA among all: *F*_(3,39)_ = 5.680, *P* = 0.003.

Then, we performed the OF task, which allows to study locomotor activity, anxiety-like and stereotyped behaviors (Kelley, 2001; Prut and Belzung, 2003). No differences were found in the % time spent in the center (*F*_(3,44)_ = 1.144, *P* = 0.342), the number of crosses (*F*_(3,44)_ = 6.656, *P* = 0.308), the horizontal activity (*F*_(3,44)_ = 2.679, *P* = 0.059), the vertical activity (*F*_(3,44)_ = 2.769, *P* = 0.053), suggesting that locomotor activity and anxiety-like behavior were not influenced by genotype and treatment (Figures 6F–I). However, *Fmr1* KO mice showed an increase of grooming (Bonferroni’s *P* < 0.0001; Figure 6J) and a decrease of freezing (Bonferroni’s *P* = 0.036; Figure 6K) compared to WT littermates that were rescued by treatment with LP-211 (Bonferroni’s *P* = 0.245 and *P* = 1). No differences were detected in defecation (Figure 6L), considered as an indirect index of anxiety.

## Discussion

We have studied the intracellular action mechanisms underlying 5-HT_7_R-mediated reversal of mGluR-LTD in WT and *Fmr1* KO mice, a model of FXS. mGluR-LTD plays a fundamental role in learning and memory (Luscher and Huber, 2010; Sanderson et al., 2016) and is abnormally enhanced in the hippocampus of *Fmr1* KO mice (Huber et al., 2002). Exaggerated hippocampal mGluR-LTD has been confirmed by several studies (Hou et al., 2006; Zhang et al., 2009; Choi et al., 2011; Costa et al., 2012a; Till et al., 2015), and is considered as a reliable readout of synaptic dysfunction in animal models of FXS and a cause of learning impairment and behavioral alterations (Sanderson et al., 2016). FMRP, the protein lacking in FXS, is most highly expressed at PN 7–12 (Davidovic et al., 2011; Bonaccorso et al., 2015). In the absence of FMRP, dendritic spine morphology and synapse formation are impaired in cortex (Comery et al., 1997) and hippocampus (Grossman et al., 2010) of *Fmr1* KO mice. We have studied the effect of 5-HT_7_R activation on mGluR-LTD at PN 14–23, a developmental stage during which physiological synaptogenesis (Semple et al., 2013) and synaptic pruning (Jawaid et al., 2018) reach the highest levels in the brain of rodents.

We have previously shown that activation of 5-HT_7_ receptors reverses mGluR-LTD in WT and *Fmr1* KO mouse hippocampus (Costa et al., 2012a). Different effects of the 5-HT_7_R agonist LP-211 were observed in mouse cerebellar cortex, where application of LP-211 induced a long-term depression of basal glutamatergic transmission in parallel fibers—Purkinje cells synapses (Lippiello et al., 2016). In the hippocampal CA3-CA1 synapse, instead, we never observed any long-term effect of LP-211 on basal synaptic transmission: application of LP-211 induced a transient enhancement of EPSC amplitude that fully recovered within 20 min (Costa et al., 2012b), indicating that 5-HT_7_ receptors activate different mechanisms in distinct brain areas.

Here, we show that the amount of hippocampal mGluR-LTD, which is abnormally enhanced in *Fmr1* KO neurons, is reduced by 5-HT_7_ receptors through an increase in intracellular cAMP levels. Our conclusion is supported by data showing that 5-HT_7_R-mediated reversal of mGluR-LTD was: (1) mimicked by forskolin, a direct stimulator of adenylate cyclase; (2) mimicked by PACAP, a potent endogenous stimulator of adenylate cyclase; (3) completely abolished by SQ 22536, an adenylate cyclase inhibitor; and (4) fully blocked by an inhibitor of PKA, one of the main cAMP target enzymes.

In the presence of an adenylate cyclase blocker or of a PKA blocker, mGluR-LTD persisted but 5-HT_7_R-mediated effect was abolished. These results indicate that the cAMP/PKA pathway is not required for mGluR-LTD induction and/or expression, in agreement with previous studies (Camodeca et al., 1999; Schnabel et al., 2001), but stimulation of this pathway by 5-HT_7_ receptors modulates the amount of mGluR-LTD.

In WT neurons, following blockade of adenylate cyclase the amount of mGluR-LTD became comparable to that observed in *Fmr1* KO slices, suggesting that exaggerated mGluR-LTD in Fmr1 KO mice might be related to reduced cAMP production. This hypothesis is in line with different studies supporting the view that the cAMP cascade is impaired in FXS. The hypothesis of low cAMP levels in FXS was suggested by early studies showing reduced basal cAMP levels and reduced cAMP production in blood platelets from FXS patients (Berry-Kravis and Huttenlocher, 1992; Berry-Kravis and Sklena, 1993). Another study shows that forskolin-induced cAMP production was reduced in blood platelets and brain tissues from different FXS animal models, but basal cAMP levels were unchanged (Kelley et al., 2007). Comparable total cAMP levels were also found in WT and *Fmr1* KO mouse hippocampus homogenates (Sethna et al., 2017). Furthermore, a very recent report reveals that the mRNA encoding phosphodiesterase 2A (PDE2A), the main cAMP degradative enzyme, is a prominent target of FMRP and the absence of FMRP leads to PDE2A overexpression in cortical and hippocampal *Fmr1* KO neurons (Maurin et al., 2018a). Overall these data strongly support the hypothesis of unbalanced cAMP production/degradation potentially leading to reduced cAMP levels in FXS.

In the present work, we show that activation of 5-HT_7_Rs reversed mGluR-LTD through an increase of intracellular cAMP levels and activation of PKA, one of the main effectors of cAMP.

5-HT_7_R activation also stimulated ERK, a subclass of the MAPKs that can interact with the cAMP pathway (Dumaz and Marais, 2005). In the hippocampus, ERK phosphorylation is stimulated by activation of group I mGluRs and is required for mGluR-LTD (Gallagher et al., 2004; Banko et al., 2006). Our electrophysiology data confirm that mGluR-LTD was completely abolished by intracellular inclusion of a MAPK/ERK blocker, thus required ERK activation. Using Western blotting, we found that activation of 5-HT_7_Rs by LP-211 also stimulated ERK phosphorylation in WT mice, with a major effect on the FVB background, in line with previous reports (Errico et al., 2001; Lin et al., 2003; Norum et al., 2003). Interestingly, we detected a much lower and not significant 5-HT7R-mediated ERK phosphorylation in *Fmr1* KO mice, in line with evidence showing that ERK phosphorylation after receptor activation is reduced or blunted in Fragile X mouse (Hou et al., 2006; Hu et al., 2008; Kim et al., 2008; Shang et al., 2009). Future experiments will be aimed at clarifying the mechanisms underlying the reduced/absent ERK phosphorylation after 5-HT_7_ receptor stimulation in *Fmr1* KO mice. For the purpose of the present study, since mGluR-LTD was significantly reversed by 5-HT_7_R activation in both WT and *Fmr1* KO mice, the lack of phospho-ERK activation in Fmr1 KO mice suggests that LP-211-mediated reversal of mGluR-LTD operates through a distinct mechanism that does not involve stimulation of ERK signaling.

Other intracellular proteins are crucially involved in mGluR-LTD among which the activity-regulated cytoskeletal-associated protein (Arc; Arc/Arg3.1). Arc/Arg3.1 belongs to the intracellular signaling machinery that is necessary for mGluR-LTD, as a rapid synthesis of Arc/Arg3.1 is triggered by mGluR activation and induces mGluR-dependent endocytosis of AMPA receptors (Park et al., 2008). Several other intracellular molecules are necessary for mGluR-LTD (Luscher and Huber, 2010), thus modulation of mGluR-LTD may occur at many different steps. We do not exclude that 5-HT_7_R activation might modulate translation/transcription of proteins playing a key role in mGluR-LTD; we are currently investigating which “LTD proteins” might be regulated by 5-HT_7_ receptors.

Our present data indicate that 5-HT_7_ receptors reverse mGluR-LTD through the cAMP/PKA pathway, which is not required for mGluR-LTD induction and/or expression as shown by previous data and confirmed by our results. As a matter of fact, inhibition of adenylate cyclase or PKA did not block mGluR-LTD but completely blocked 5-HT_7_-mediated reversal of mGluR-LTD. Thus, the 5-HT_7_R-activated pathway is not necessary for mGluR-LTD induction but is able to modulate the final amount of synaptic inhibition.

We have previously speculated that activation of 5-HT_7_ receptors may indeed affect AMPA receptor trafficking. Interestingly, 5-HT_7_R activation was recently shown to phosphorylate AMPA receptors in rat hippocampal neurons, increasing their membrane insertion and conductance, thus enhancing AMPAR-mediated synaptic currents (Andreetta et al., 2016), consistent with data from our laboratory (Costa et al., 2012b). We have also shown that 5-HT_7_ receptor activation prevented DHPG-induced internalization of AMPA receptors in WT and *Fmr1* KO (Costa et al., 2012a). In view of these data, the 5-HT_7_R-activated cAMP/PKA pathway might ultimately phosphorylate AMPA receptors, reducing their internalization and increasing their conductance, thus reducing the amount of mGluR-LTD.

Our result that increasing cAMP levels rescues abnormal synaptic plasticity in *Fmr1* KO hippocampus suggests that other molecules acting on Gs-coupled receptors might be used for FXS therapy. Among these, we show that PACAP is a promising candidate since this neurotrophic peptide modulates hippocampal synaptic transmission (Costa et al., 2009) and plasticity, correcting abnormal mGluR-LTD in *Fmr1* KO neurons (present results).

In accordance with cAMP involvement, exaggerated mGluR-LTD in *Fmr1* KO neurons was reversed by mGluR2 blockade (Choi et al., 2011, 2016) or by PDE4 inhibition (Choi et al., 2015, 2016), both virtually increasing cAMP levels. Altered mechanisms leading to reduced cAMP production in FXS are under investigation. Over-expression of FMRP causes an increased production of cAMP in transfected cell lines, leading to the conclusion that FMRP might directly regulate the translation of mRNA(s) coding for cAMP cascade proteins (Berry-Kravis and Ciurlionis, 1998). Indeed, several mRNAs encoding components of cAMP signaling cascade are target of FMRP (Darnell et al., 2011) among which, as above discussed, PDE2A is a major FMRP target (Maurin et al., 2018a). Importantly, very recent data demonstrate that PDE2A is overactivated in *Fmr1* KO mouse brain, leading to reduced cAMP levels, and pharmacological inhibition of PDE2A reverses exaggerated mGluR-LTD in *Fmr1* KO hippocampus (Maurin et al., 2018b).

Impaired signaling through dopamine Gs-coupled receptors was also evidenced in *Fmr1* KO cultured cortical neurons, where D1 receptor-stimulated cAMP formation was decreased with respect to WT, due to reduced coupling of D1 receptors to adenylate cyclase (Wang et al., 2008). Interestingly, the same work also shows that AS-19, a selective 5-HT_7_R agonist, instead stimulated adenylate cyclase comparably in WT and in *Fmr1* KO neurons and we show that 5-HT_7_R activation corrects excessive mGluR-LTD in *Fmr1* KO mice. These results have important therapeutic implications indicating that, unlike D1 receptors, 5-HT_7_Rs are fully functional in *Fmr1* KO mice, thus 5-HT_7_R agonists might become new pharmacological tools.

In this perspective, in this manuscript we also investigated whether systemic administration of LP-211 to *Fmr1* KO might rescue learning and behavioral deficits that mirror cognitive impairment and autistic-like behavior in FXS patients. We used the NOR test to study cortex- and hippocampus-dependent novelty detection ability (Broadbent et al., 2010), which is known to involve hippocampal mGluR-LTD (reviewed by Sanderson et al., 2016). We first confirmed that recognition memory tested by NOR is impaired in *Fmr1* KO mice (Ventura et al., 2004; King and Jope, 2013; Franklin et al., 2014; Gomis-González et al., 2016), since discrimination index is impaired compared to WT littermates and less time is spent exploring the novel object. Interestingly, *Fmr1* KO mice showed a preference for the familiar compared to the novel object, consistent with previous studies demonstrating alterations of novelty preferences, with stereotyped behavior and restricted interests, in autism spectrum disorders (Jacob et al., 2009). Here we show that an acute systemic administration of LP-211 rescued recognition memory impairment in *Fmr1* KO mice.

About one third of FXS patients display autistic behavior, including gaze and touch avoidance and repetitive behavior (Garber et al., 2008). Using the marble burying and the OF tasks, two protocols revealing stereotyped behavior in rodents (Thomas et al., 2009), *Fmr1* KO mice showed increased repetitive behavior, i.e., marble burying and grooming, with respect to WT, as previously demonstrated (Veeraragavan et al., 2012; Gholizadeh et al., 2014; Kazdoba et al., 2014). Interestingly, *Fmr1* KO mice presented less spontaneous freezing behavior compared to WT, consistent with previous studies in GAP43 mice model of autism spectrum disorder (Zaccaria et al., 2010) and probably reflecting some aspects of maladaptive behavior to stress and catatonia in patients. This phenotype was completely rescued by systemic administration of LP-211.

In conclusion, we show that selective activation of 5-HT_7_Rs corrects abnormal intracellular signaling and synaptic plasticity in newborn *Fmr1* KO mice and rescues learning and behavior in young adult *Fmr1* KO mice. The latter result has important implications for therapy, indicating that a rescue of FXS phenotypes by pharmacological treatment can also be possible at adult age. Therefore, selective 5-HT_7_ receptor agonists might represent a new pharmacological strategy for FXS therapy.

## Author Contributions

LCosta: electrophysiology data collection, analysis and interpretation; final approval of manuscript. LS: electrophysiology data collection; animal care, final approval of manuscript. MS, CB and SD’A: western blotting data collection, analysis and interpretation; animal care, final approval of manuscript. WG and MT: behavioral data collection, analysis and interpretation; final approval of manuscript. ML and EL: design and synthesis of 5-HT_7_R agonists; data interpretation; final approval of manuscript. LCiranna, MVC and DP: conception and design; data analysis and interpretation; manuscript writing; final approval of manuscript.

## Conflict of Interest Statement

The authors declare that the research was conducted in the absence of any commercial or financial relationships that could be construed as a potential conflict of interest.

## Supplementary Material

FIGURE S1 LP-211 treatment increased levels of both phospho-ERK1 and phospho-ERK2 in hippocampal slices of wild-type (WT) mice. **(A)** Semi-quantitative analysis of phosphorylated ERK1 vs. total ERK1 in control and LP-211 (10 nM, 5 min) treated hippocampal slices from WT mice (FVB strain). Relative optical density is presented as percentage of control. Data represent mean ± SEM of four separate experiments, each performed on a pool of three mice. **p* = 0.0286 by Mann-Whitney Rank Sum Test. **(B)** Semi-quantitative analysis of phosphorylated ERK2 vs. total ERK2 in control and LP-211 treated hippocampal slices from WT mice (FVB strain). Relative optical density is presented as percentage of control. Data represent mean + SEM of four separate experiments, each performed on a pool of three mice. **p* = 0.0412 by unpaired *t*-test.

FIGURE S2 Original images of immunoblots shown in Figure 5A.

FIGURE S3 **(A)** Original images of immunoblots shown in Figure 5B. **(B)** Original images of immunoblots shown in Figure 5C.

FIGURE S4 Experimental procedures for marble burying test. Marbles distribution before testing shows twenty marbles equidistantly distributed. Mice were left in the cage for 20 min and number of buried marbles was analyzed in four different groups (vehicle-treated WT; WT treated with LP-211; vehicle-treated *Fmr1* KO; *Fmr1* KO treated with LP-211).
