# Supplementary figures and images for: Activation of Serotonin 5-HT7 Receptors Modulates Hippocampal Synaptic Plasticity by Stimulation of Adenylate Cyclases and Rescues Learning and Behavior in a Mouse Model of Fragile X Syndrome
Source: Front Mol Neurosci. 2018 Oct 2;11:353. doi: 10.3389/fnmol.2018.00353 (PMC6176069; doi:10.3389/fnmol.2018.00353)

Supplementary Fig. 1

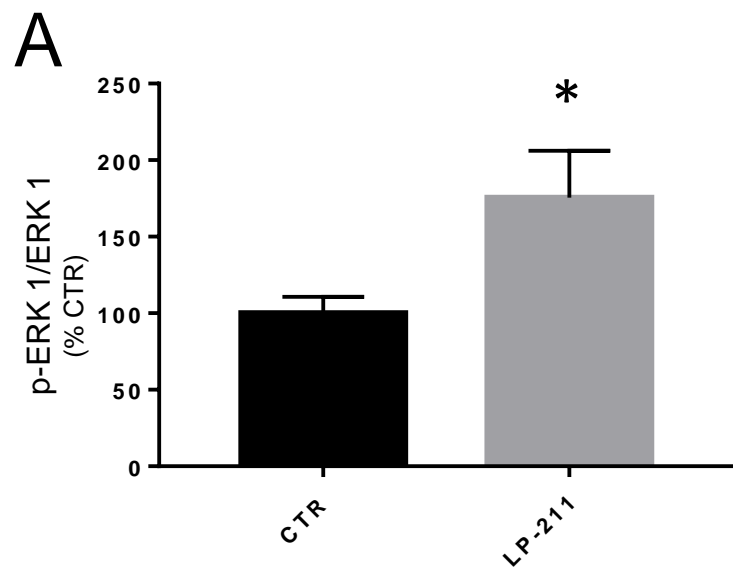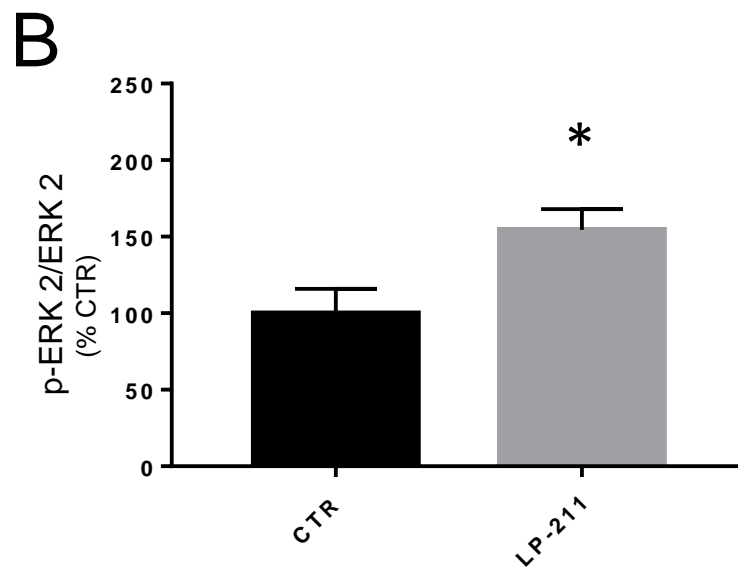

Supplement: FIGURE S1 — LP-211 treatment increased levels of both phospho-ERK1 and phospho-ERK2 in hippocampal slices of wild-type (WT) mice. (A) Semi-quantitative analysis of phosphorylated ERK1 vs. total ERK1 in control and LP-211 (10 nM, 5 min) treated hippocampal slices from WT mice (FVB strain). Relative optical density is presented as percentage of control. Data represent mean ± SEM of four separate experiments, each performed on a pool of three mice. *p = 0.0286 by Mann-Whitney Rank Sum Test. (B) Semi-quantitative analysis of phosphorylated ERK2 vs. total ERK2 in control and LP-211 treated hippocampal slices from WT mice (FVB strain). Relative optical density is presented as percentage of control. Data represent mean + SEM of four separate experiments, each performed on a pool of three mice. *p = 0.0412 by unpaired t-test. [file Image_1.PDF]

Wild-type  
(FVB)

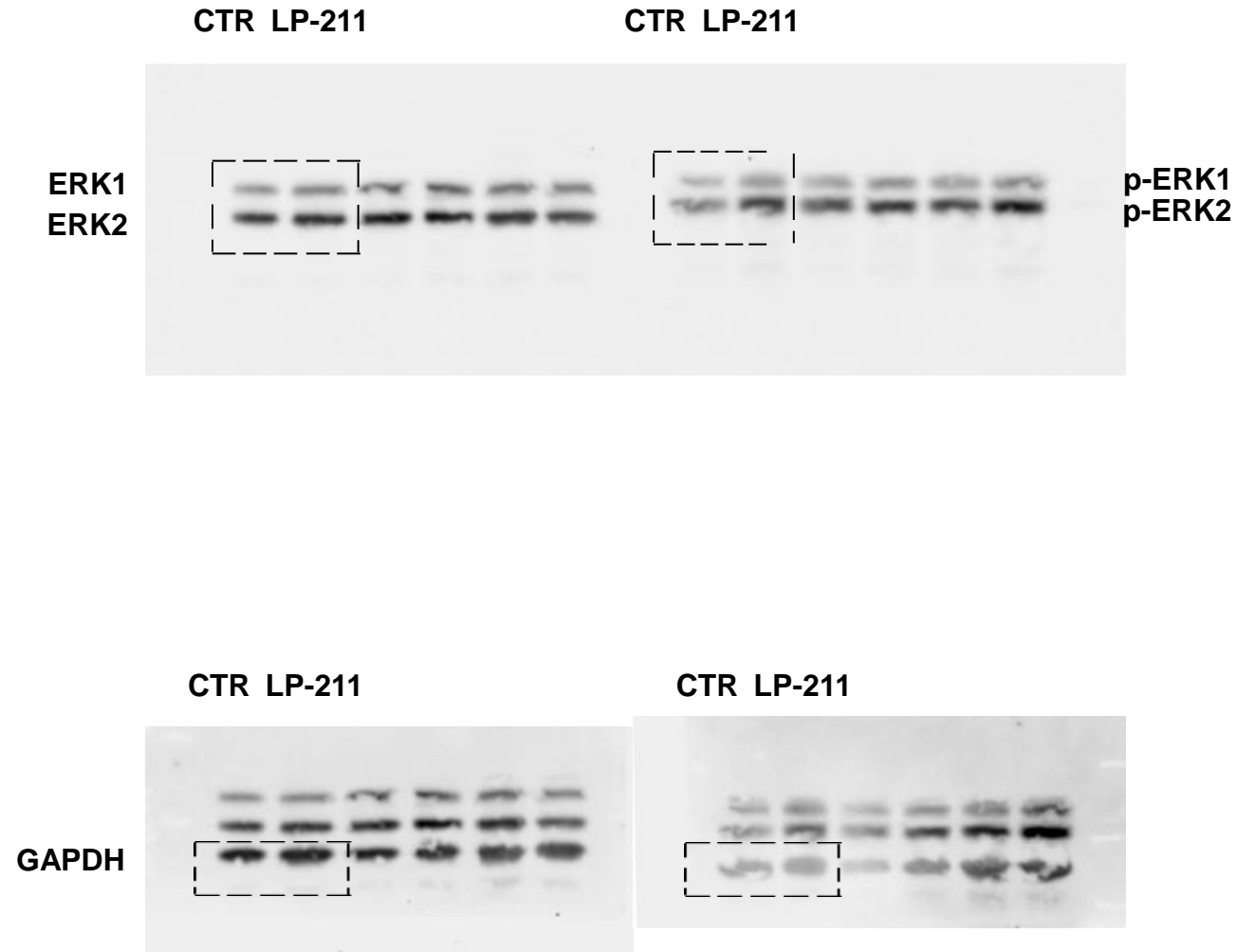

Supplement: FIGURE S2 — Original images of immunoblots shown in Figure 5A. [file Image_2.PDF]

**A**

Wild-type  
(C57BL/6J)

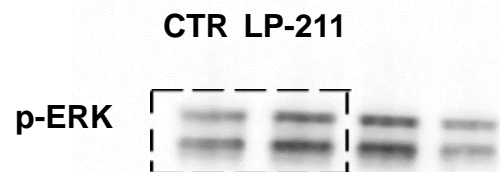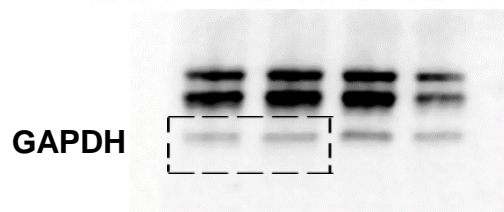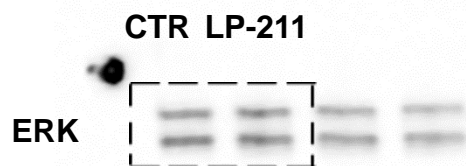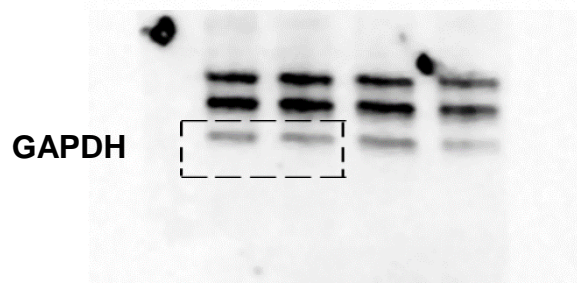

**B**

*Fmr1* KO  
(C57BL/6J)

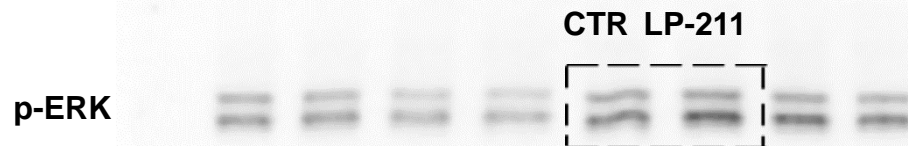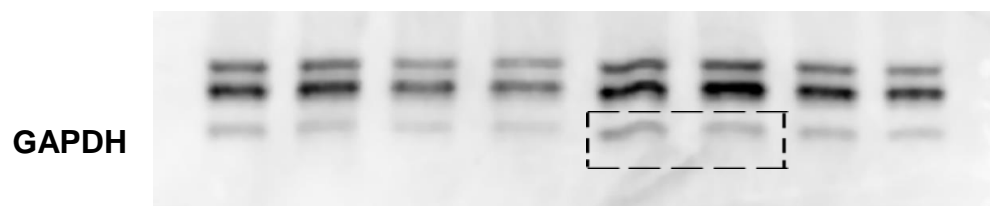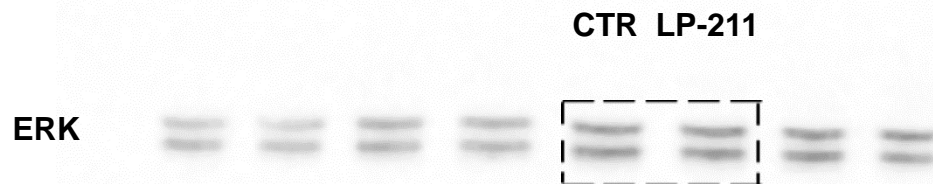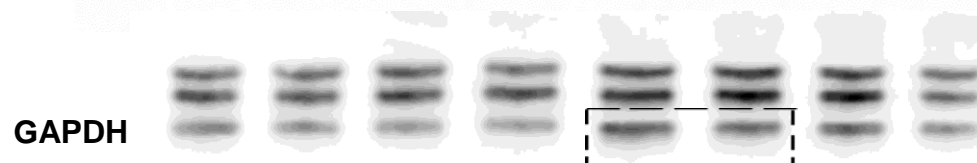

Supplement: FIGURE S3 — (A) Original images of immunoblots shown in Figure 5B. (B) Original images of immunoblots shown in Figure 5C. [file Image_3.PDF]

Supplementary Fig. 4

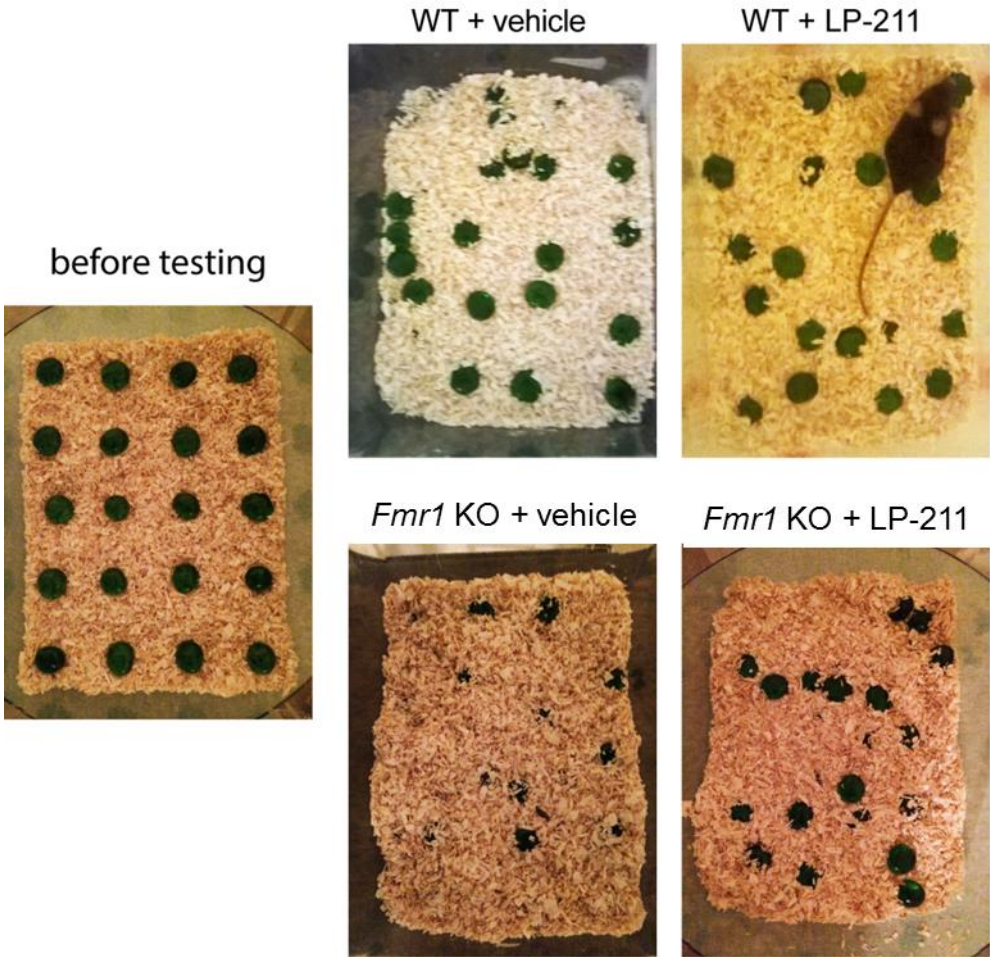

Supplement: FIGURE S4 — Experimental procedures for marble burying test. Marbles distribution before testing shows twenty marbles equidistantly distributed. Mice were left in the cage for 20 min and number of buried marbles was analyzed in four different groups (vehicle-treated WT; WT treated with LP-211; vehicle-treated Fmr1 KO; Fmr1 KO treated with LP-211). [file Image_4.PDF]
